# Comprehensive analysis of aging-related genes and immune infiltration landscape in ischemic cardiomyopathy

**DOI:** 10.3389/fcvm.2025.1653314

**Published:** 2025-09-11

**Authors:** Zhichao Wu, Liang Du, Xunfu Zhang, Chengyu Jin, Jinshan Ma

**Affiliations:** ^1^ Graduate school, Xinjiang Medical University, Urumqi, Xinjiang, China; ^2^ Department of Thoracic Surgery, People’s Hospital of Xinjiang Uygur Autonomous Region, Urumqi, Xinjiang, China

**Keywords:** aging, bioinformatic analysis, ischemia-reperfusion injury, ischemic cardiomyopathy, oxidative stress, inflammation

## Abstract

**Background:**

Ischemic cardiomyopathy (ICM), which arises from obstructive coronary artery diseases, is a leading cause of heart failure. Aging is a major risk factor for cardiovascular diseases, yet its connection to ICM remains unclear. This study aimed to investigate the role of aging-related gene expression in the context of ICM.

**Methods:**

Human microarray data (GSE116250) were retrieved from the GEO database and aging-related differentially expressed genes (ARDEGs) were identified using the Aging Atlas database. Functional enrichment and protein-protein interaction (PPI) network analyses were performed to elucidate the functions and interactions of these ARDEGs, leading to the identification of hub genes. The immune infiltration landscape in ICM was further characterized. Subsequently, integrated regulatory networks involving the hub genes were constructed through microRNA-mRNA-transcription factor interaction and GeneMANIA analyses, while their biological functions were inferred via gene set enrichment analysis (GSEA). The predictive value of the hub genes was validated using receiver operating characteristic (ROC) analysis based on both the identification dataset (GSE116250) and an independent validation cohort (GSE1145) Finally,the expression patterns of these genes were verified by RT-qPCR on mouse disease model.

**Results:**

A total of 50 ARDEGs (42 upregulated and 8 downregulated) were identified,which are primarily involved in the response to inflammation. Ten types of immune cells showed significant alterations in the ICM heart tissues. Among these cells, CD56dim NK cells exhibited extensive and significant correlations with other immune cells. JUN was identified as a key transcription factor regulating the top five hub ARDEGs: TNF, PTGS2, IL6, IL1B, and CXCL8. ROC analysis demonstrated that TNF, CXCL8, and IL6 serve as potential biomarkers for ICM, and the combination of the three markers further improved the predictive value. RT-qPCR analysis subsequently confirmed the upregulation of these hub inflammatory ARDEGs in mouse heart tissues.

**Conclusion:**

Aging-related genes play a significant role in ICM and targeting these genes may pave the way for ICM diagnostic and therapeutic strategies.

## 1 Introduction

Ischemic cardiomyopathy (ICM), the terminal stage of coronary heart disease, mainly presents as impaired left ventricular systolic function and is the leading cause of heart failure (HF) globally. Given that cardiomyocytes lack proliferative capacity, obstructive coronary artery diseases, especially acute myocardial infarction (MI), usually lead to irreversible cardiac function loss. Severe MI causes extensive cardiomyocyte death, which triggers fibroblast infiltration and migration, ultimately resulting in excessive fibrosis, further limiting the cardiac function of the ischemic heart and leading to adverse clinical outcomes. Thus, understanding the underlying mechanisms of ICM occurrence and development is crucial for its prevention and treatment.

Aging is an inevitable human life process, characterized by genomic instability, telomere attrition, epigenetic alterations, proteostasis loss, macroautophagy impairment, nutrient-sensing deregulation, mitochondrial dysfunction, cellular senescence, stem cell exhaustion, altered intercellular communication, chronic inflammation, and dysbiosis (1). Aging-related genes (ARGs) play a significant role throughout the aging process; experimentally, enhanced ARG expression accelerates aging, while interventions on them can reverse aging. To date, 503 ARGs have been identified in the Aging Atlas database, which are closely related to various clinical diseases, including cardiovascular diseases.

Aging not only increases the prevalence of obstructive coronary diseases but is also associated with a higher incidence of heart failure and death following myocardial infarction (2, 3). On one hand, coronary artery atherosclerosis (AS), the pathological basis of obstructive coronary artery diseases, accelerates with age. A mature atherosclerotic lesion is marked by the aggregation of vascular smooth muscle cells (VSMCs), along with their extracellular matrix contributions, mainly collagen and elastin. This environment also contains inflammatory cells such as T lymphocytes, dendritic cells, macrophages, and mast cells. Intracellular and extracellular lipids, along with debris, accumulate within a central necrotic core, which is surrounded by a fibrous covering mainly composed of VSMCs. The stability of the atherosclerotic plaque is determined by the firmness of this fibrous covering and its inflammatory activity level. Aging affects endothelial cell (EC) function, alters VSMC proliferation, and promotes inflammatory cell infiltration, ultimately leading to atherosclerotic plaque formation and rupture. On the other hand, in cardiomyocytes, aged cardiomyocytes show lower tolerance to ischemic and hypoxic conditions compared to younger cells, mainly due to impaired mitochondrial function and disrupted energy metabolism (4). These alterations render aged cardiomyocytes more susceptible to apoptosis (2). After myocardial infarction, the death of these cells can lead to rapid and substantial myocardial tissue loss, severely compromising cardiac function and ultimately resulting in ICM. Therefore, investigating aging-associated genes in the context of ICM may yield promising therapeutic targets and diagnostic markers for ICM management. However, the interplay between aging and ICM and its operational mechanism still requires further exploration.

In the present study, bioinformatic analysis techniques were used to profile immune infiltration landscape, identify aging-associated genes in ICM and elucidate the underlying mechanisms linking these genes to ICM progression. A set of hub regulatory genes was identified and subsequently validated through a combination of bioinformatic approaches and RT-qPCR experiments on animal models. These findings provide a crucial direction for future experimental investigations, potentially enhancing risk-assessment-based preventive measures and therapeutic strategies for ICM.

## 2 Materials and methods

### 2.1 Data collection

The datasets utilized in this study are publicly accessible through the Gene Expression Omnibus (GEO) repository (https://www.ncbi.nlm.nih.gov/geo/), a comprehensive public database of high-throughput functional genomic data, with an accession number of GSE116250 for the differentially expressed genes (DEGs) identification dataset. The GEO dataset GSE1145 was used as a validation analysis dataset. The GSE116250 dataset contained 14 normal heart tissues and 13 ICM tissues. The GSE1145 dataset contained 11 normal control heart samples and 20 ICM heart tissues. The ARGs were acquired from the Aging Atlas database (https://ngdc.cncb.ac.cn/aging/age_related_genes) which involved 503 entries.

### 2.2 Determination of DEGs and ARDEGs

GEO2R, an online tool (http://www.ncbi.nlm.nih.gov/geo/geo2r/), was employed to screen DEGs between normal heart and ICM heart samples. In the present study, genes with a *P* (adj)-value <0.05 and |log (fold change) (FC)| > 1 were deemed as DEGs. The Venn Diagram tool (accessible at http://bioinformatics.psb.ugent.be/webtools/Venn/), was employed to find the overlap between selected DEGs and ARGs from aging databases, thereby revealing critical age-related differentially expressed genes (ARDEGs). ARDEGs were visualized using a heatmap based on the mean and standard deviation (SD) of the series matrix data from samples.

### 2.3 GO and KEGG pathway analysis

The Gene Ontology (GO) analysis and Kyoto Encyclopedia of Genes and Genomes (KEGG) analysis were conducted for the ARDEGs. The GO is an international standardized gene function classification system which is composed of three categories: biological process (BP), cell component (CC), as well as molecular function (MF). The KEGG analysis was applied to determine related signaling pathways for ARDEGs. Both GO and KEGG were analyzed and visualized by the R package “clusterProfiler” and “pathview”.

### 2.4 PPI network construction and hub gene selection

The STRING database (https://cn.string-db.org/) was utilized to access ARDEG-encoded proteins and PPI information. The PPI network was subsequently constructed by using Cytoscape software 3.9.1. The MCODE plug-in was used to perform modular analysis, and the most significant module was identified based on the MCODE score and node number.

### 2.5 Immune infiltration analysis

Single-sample Gene Set Enrichment Analysis (ssGSEA) was utilized to analyze the degree of immune cell infiltration in control and ICM group. A heatmap was used to exhibit the differences in immune cells between the samples of the two groups. Differential abundance of these immune cells between the two groups was assessed using Wilcoxon rank-sum tests,with results presented in boxplots generated by ggplot2R package. Spearman correlation analysis implemented in the “psych” R package was subsequently performed to construct interaction networks among differentially abundant immune cells and evaluate associations between hub ARDEGs and altered immune cell subsets. A correlation coefficient threshold (cor) >0.3 and *P* < 0.05 was accepted in this analysis.

### 2.6 Gene set enrichment analysis (GSEA)

As an advanced analytical method that applies molecular feature databases, Gene Set Enrichment Analysis (GSEA) employs a precise algorithm to interpret the effects of gene expression. For each hub gene, genome-wide Spearman correlation coefficients with all other genes were computed using the R package “psych”. Genes were then ranked by descending correlation coefficient to generate biomarker-specific gene lists. GSEA was performed against the “c2.cp.kegg_medicus.v2025.1.Hs. Symbols” reference set from MSigDB using the clusterProfiler R package, with significantly enriched pathways identified at thresholds of |NES| > 1 and nominal *p*-value < 0.05.

### 2.7 GeneMANIA analysis

GeneMANIA (http://genemania.org) is a comprehensive online tool that integrates multiple data sources including physical interactions, co-expression, co-localization, genetic interactions, pathway enrichment, and literature-based predictions. In this study, GeneMANIA was used to predict and construct a functional network.

### 2.8 Transcription factors (TFs)-mRNA-miRNAs regulatory network analysis

To further identify the underlying regulators of hub-ARDEGs, transcription factors were predicted by using the TRRUST database (https://www.grnpedia.org/trrust/), and miRNA of hub-ARDEGs were predicted by both using the miRWalk database (http://mirwalk.umm.uni-heidelberg.de/) and miRDB database (https://mirdb.org/). The predicted TFs were then overlaped with GSE116250, miRNAs were overlapped between the two databases. The intersecting TFs and miRNAs were identified and retained. Cystoscope software (3.9.1) was utilized to visualize the relevant results.

### 2.9 Correlation analysis among the hub ARDEGs

To evaluate the relationships among these hub ARDEGs, the correlations among the hub ARDEGs were conducted by using Pearson correlation analysis. In the Pearson's correlation analysis,the r value refers to the correlation coefficient and was used to evaluate the effect size. And then the correlation matrix heatmap and the scatter plots were mapped by using the R package “ggplot2”.

### 2.10 ROC analysis of the hub ARDEGs

The gene expression of the corresponding hub ARDEGs was obtained in the GSE116250 and GSE1145 datasets. Receiver operating characteristics (ROC) analysis was applied to obtain the area under the curve (AUC) values by using the “pROC” package (v 1.18.5). In the ROC curve, sensitivity values were plotted on the *Y*-axis, and the false positive rates (1-specificity) values were plotted on the *X*-axis. The ROC curve with an AUC value ≥0.70 was considered to indicate an adequate predictive value.

### 2.11 Nomogram establishment

To evaluate the diagnostic capability of the ARDEGs with an AUC value ≥0.70 in both GSE116250 and GSE1145, a diagnostic nomogram was constructed using the rms package (v6.5-0). The nomogram included both individual and total points, where higher total points corresponded to a higher likelihood of ICM. The nomogram's effectiveness was evaluated using a calibration curve generated with the “rms”R package,the Hosmer–Lemeshow (HL) test and ROC curve analysis, generated with the rms package and pROC package (v1.18.5), respectively. The calibration curve closely matching the ideal curve, an HL test *p*-value greater than 0.05, and an ROC curve AUC greater than 0.7 indicated that the nomogram had strong predictive capability.

### 2.12 Animal study

The experimental myocardial infarction model was developed in aged male C57BL/6 mice (12 months old). The mice were obtained from the animal center of Xinjiang Medical University. All animals were maintained in a pathogen-free environment. Commercial chow and tap water were made available *ad libitum*. All animals received humane care in compliance with the “Principles of Laboratory Animal Care” formulated by the National Society for Medical Research and the “Guide for the Care and Use of Laboratory Animals” prepared by the Institute of Laboratory Animal Resources and published by the National Institutes of Health (NIH Publication No. 86-23, revised 1996). This study was approved by the Ethics Committee of the People's Hospital of Xinjiang Autonomous Region.

Twelve mice were randomly divided into two groups: the sham-operated group (Sham, *n* = 5) and the myocardial infarction group (MI, *n* = 7). Anesthesia was induced by pentobarbital sodium (50 mg/kg) i.p. injection. A 22-gauge intravenous catheter was used for tracheal intubation to facilitate mechanical ventilation, ensuring stable respiration during the procedure. The MI model was created by ligating the coronary artery. Specifically, the left anterior descending (LAD) coronary artery was carefully identified by retracting the left auricle. The LAD was permanently ligated with a 7-0 silk suture to induce myocardial infarction. The chest was closed with 7-0 silk sutures, and mice were monitored closely as they recovered. Echocardiography (VisualSonics Vevo 2,100 Imaging System, with 15 MHz probe) was used to verify the successful construction of the MI model before and 7 days post-surgery. Briefly, mice were placed supine on an electrical heating pad at 37°C under a low dose of isoflurane anesthesia for echo examination. Two-dimensional targeted M-mode traces were obtained in the position of perpendicular left ventricular (LV) anterior and posterior walls. LV internal diameter during diastole (LVDd) and LV internal diameter during systole (LVDs) were measured from M-mode recording. EF values were calculated with the formula EF = (LVDd^3^ − LVDs^3^)/LVDd^3^ × 100%. LV minor axis FS was determined as [(LVDd − LVDs)/LVDd] × 100%. Heart samples were collected from both groups after echo examination for subsequent analysis.

### 2.13 Gene expression analysis

Heart samples were harvested from animal study. Total RNA was extracted and purified from the left ventricle using RNA mini kit (12183018A, PureLink) and the concentration was quantified spectrophotometrically. Total RNA was reverse transcribed to cDNA using the Evo M-MLV Mix Kit with gDNA Clean for qPCR (AG11728, Accurate Biology). According to the manufacturer's instructions. Quantitative real-time PCR was performed on a CFX96 Real-Time PCR Detection System (Bio-Rad). The total volume of 20 ul was set including 10 ul SYBR Green PCR SuperMix (1725124, Bio-Rad), 1 ul gene-specific primer (Supplementary Table S1), 15 ug template cDNA, and RNase-free water. Transcript levels were normalized to the expression of Gapdh. Relative gene expression was assessed using the comparative *ΔΔ*CT method.

### 2.14 Statistical analysis

All results were expressed as the mean ± SD. Comparisons between the two groups were performed by using *T*-test analysis in GraphPad Prism (9.5.0). A *p*-value <0.05 was considered statistically significant.

## 3 Result

### 3.1 Determination of the aging related differentially expressed genes

**Figure 1:**
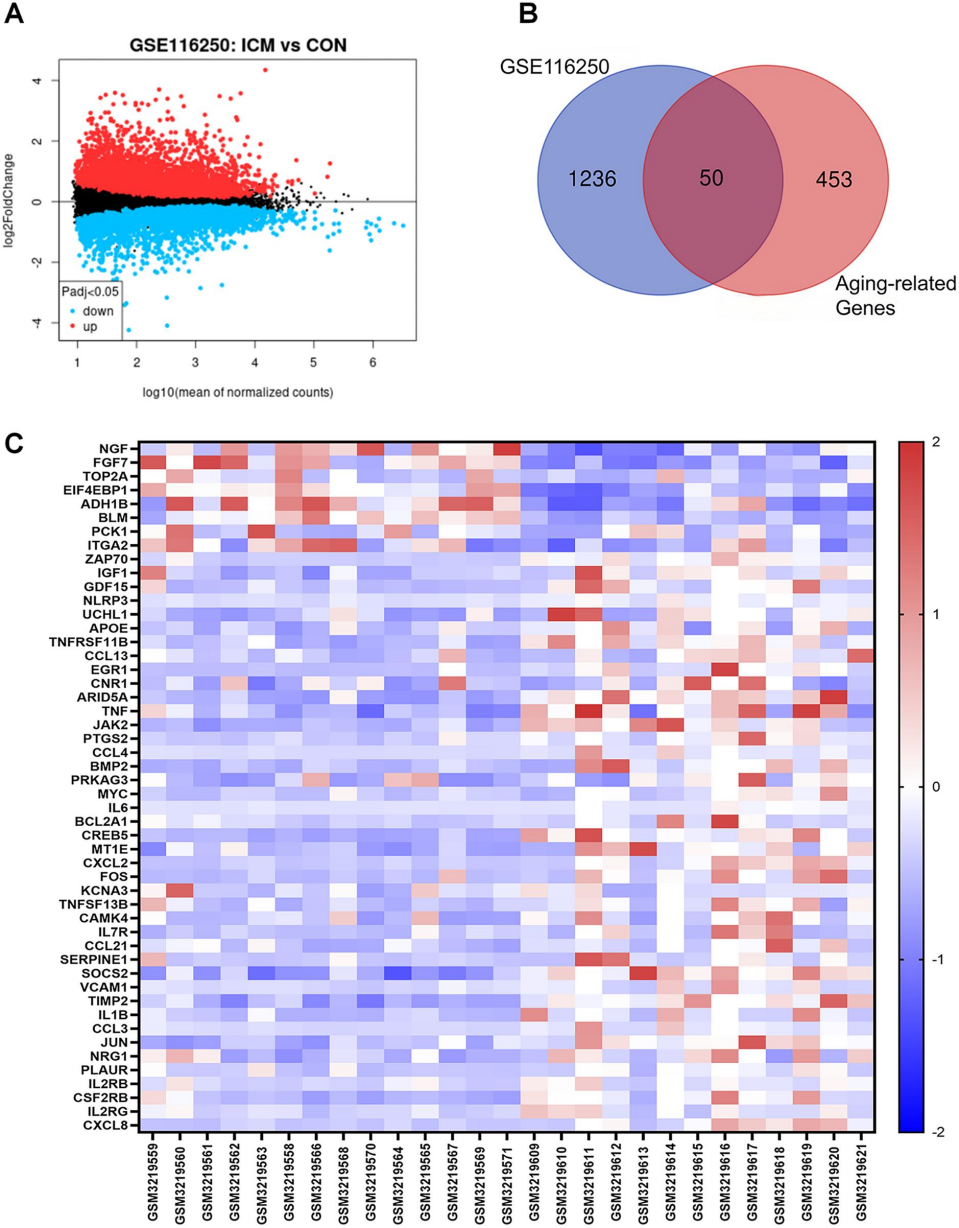
Identification of the differentially expressed genes between ICM and control groups. **(A)** The volcano plot of the differentially expressed genes in GSE116250 microarray data from the GEO database, in which the *X*-axis refers to the -log10(*p* value) and the *Y*-axis refers to the log2(fold change). The red spots represent the upregulated genes, and the blue spots represent the downregulated genes; the black spots represent the genes without significant differential expression. **(B)** Venn diagram of the differentially expressed genes analyzed according to GSE116250 and the aging-related genes based on Aging Atlas database. **(C)** The hierarchical cluster heatmap of the 50 aging related differentially expressed genes (ARDEGs). The color scale indicates the relative gene expression of each sample. The red represents upregulated genes in ICM group compared to control, and the blue represents downregulated genes in ICM group.

Based on the GSE116250 dataset, we initially screened out the differentially expressed genes (DEGs) in ICM compared to the control. The volcano plot showed a total of 1,286 DEGs, including 432 downregulated and 854 upregulated genes (Figure 1A). Subsequently, the DEGs in ICM were intersected with the aging-related genes from the Aging Atlas database. A total of 50 aging-related differentially expressed genes (ARDEGs) were identified, as shown in the Venn diagram (Figure 1B). Among these 50 ARDEGs, 8 were downregulated and 42 were upregulated, as presented in Table 1. The hierarchical cluster heatmap demonstrated that the expression of aging-related genes in the ICM group was significantly different from that in the control group (Figure 1C).

**Table 1:** A list of the 50 aging related differentially expressed genes (ARDEGs).

Gene symbols	Expression	Number
ZAP70,IGF1,GDF15,NLRP3,UCHL1,APOE,TNFRSF11B,CXCL8,EGR1,CNR1,ARID5A,TNF,JAK2,PTGS2,CCL4, BMP2,PRKAG3,MYC,IL6,BCL2A1,CREB5,MT1E,CXCL2,FOS,KCNA3,TNFSF13B,CAMK4,IL7R,CCL21,SERPINE1,SOCS2,VCAM1,TIMP2,IL1B,CCL3,JUN,NRG1,PLAUR, IL2RB,CSF2RB,IL2RG,CCL13	Upregulated	42
NGF,FGF7,TOP2A,EIF4EBP1,ADH1B,BLM,PCK1,ITGA2	Downregulated	8

### 3.2 GO and KEGG function analysis of the ARDEGs

**Figure 2:**
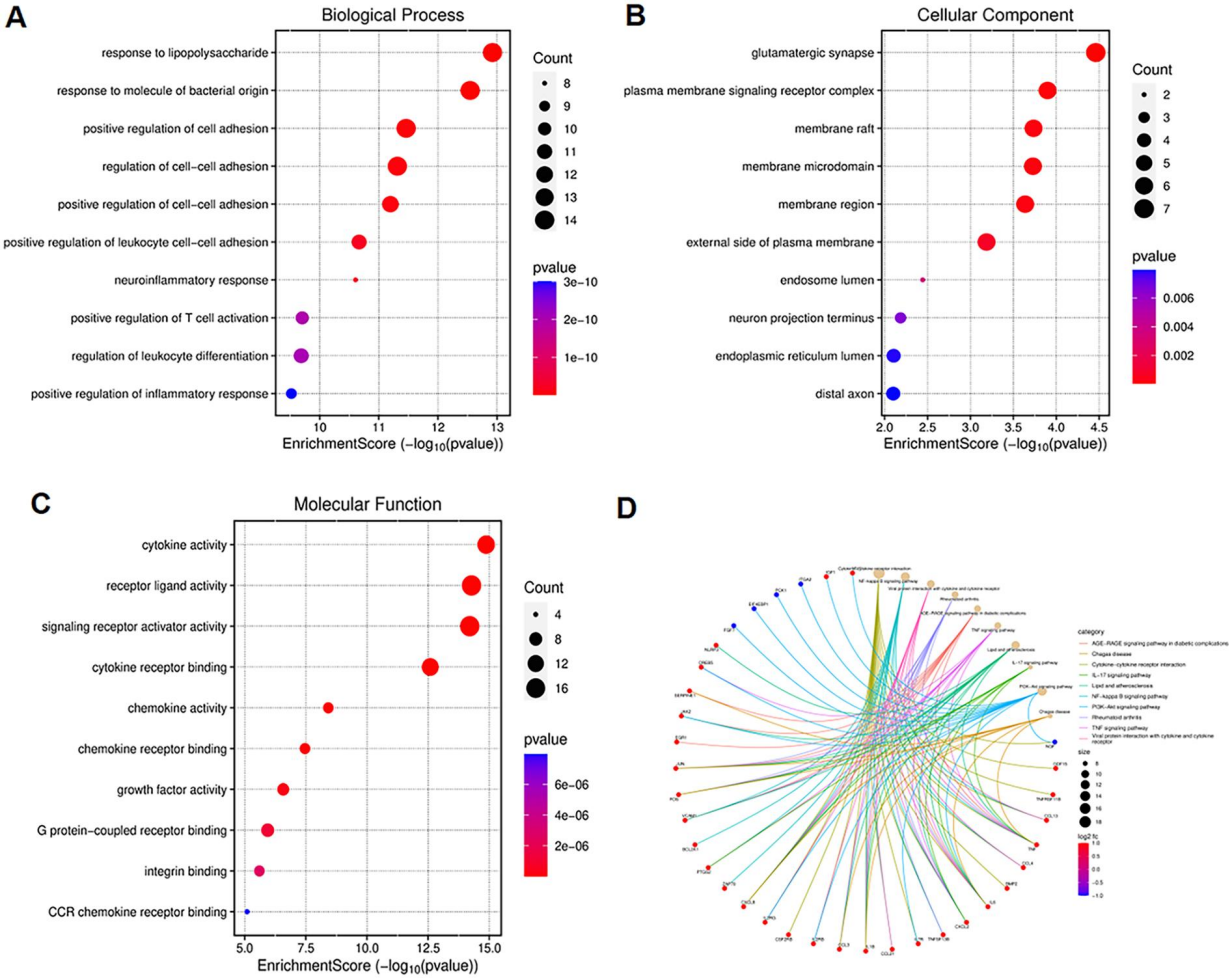
Functional enrichment analysis of the ARDEGs. **(A)** The top 10 significantly enriched GO terms in the category of biological process (BP) for the ARDEGs. **(B)** The top 10 significantly enriched GO terms in the category of cellular component (CC). **(C)** The top 10 significantly enriched GO terms in the category of molecular function (MF). **(D)** Kyoto Encyclopedia of Genes and Genomes (KEGG) pathway enrichment analysis for the ARDEGs.

The Gene Ontology (GO) is an international standardized gene function classification system commonly used to classify predicted gene functions. It has three main categories: biological process (BP), cellular component (CC), and molecular function (MF). For the GO enrichment analysis of the biological process, these ARDEGs were mainly involved in responses to lipopolysaccharide, responses to bacterial-origin molecules, and positive regulation of cell adhesion (Figure 2A). As shown in Figure 2B, the most significantly enriched GO items for the cellular component were glutamatergic synapse, plasma membrane signaling receptor complex, and membrane raft. For the molecular function, these ARDEGs were mainly involved in cytokine activity, receptor ligand activity, and signaling receptor activator activity (Figure 2C). Subsequently, we conducted Kyoto Encyclopedia of Genes and Genomes (KEGG) analysis for the 50 ARDEGs. As indicated in Figure 2D, the results showed that the ARDEGs were primarily involved in the following significant pathways: cytokine-cytokine receptor interaction, IL17 signaling pathway, NF-κB signaling pathway, PI3K-AKT signaling pathway, and TNF signaling pathway.

### 3.3 PPI analysis of ARDEGs

**Figure 3:**
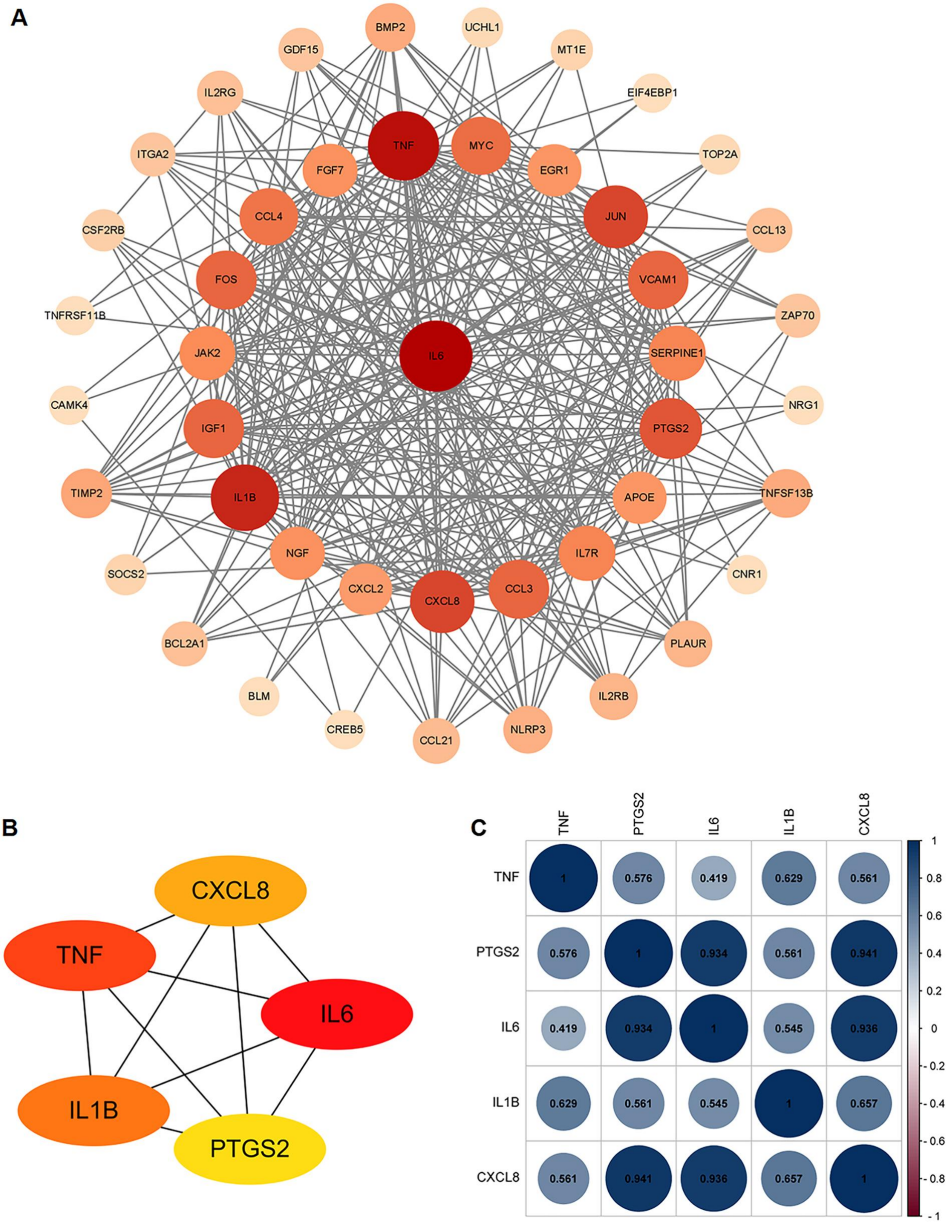
PPI analysis of the ARDEGs. **(A)** PPI analysis of these ARDEGs. **(B)** The top 5 hub ARDEGs. **(C)** Correlation analysis among the top 5 hub ARDEGs by Pearson analysis. The blue color represents positive correlation, the red color represents negative correlation.

The PPI network analysis was performed based on the STRING database and visualized using Cytoscape software (Figure 3A). Then, we determined the top five hub ARDEGs based on the PPI score, including IL6, TNF, IL1B, CXCL8, and PTGS2, as shown in Figure 3B. Detailed information on these hub ARDEGs was presented in Table 2. Interestingly, all five of these hub genes were up-regulated in ICM and had a promoting effect on the aging process. Next, we performed Pearson correlation analysis to evaluate the relationships among these hub ARDEGs (Figure 3C). All the hub genes appeared to be positively correlated with each other, and the most positively correlated pair was CXCL8-PTGS2, with an r-value of 0.941 and a *P*-value <0.01.

**Table 2:** A detailed information of the top 5 hub ARDEGs.

Gene symbols	Gene full names	Expression	Log2 (fold change)	*P* value
IL6	interleukin 6	Upregulated	2.20	<0.001
TNF	tumor necrosis factor	Upregulated	2.74	<0.001
IL1B	interleukin 1 beta	Upregulated	2.67	<0.001
CXCL8	C-X-C motif chemokine ligand 8	Upregulated	2.43	<0.001
PTGS2	prostaglandin-endoperoxide synthase 2	Upregulated	2.37	<0.001

### 3.4 Results of immune cell infiltration

**Figure 4:**
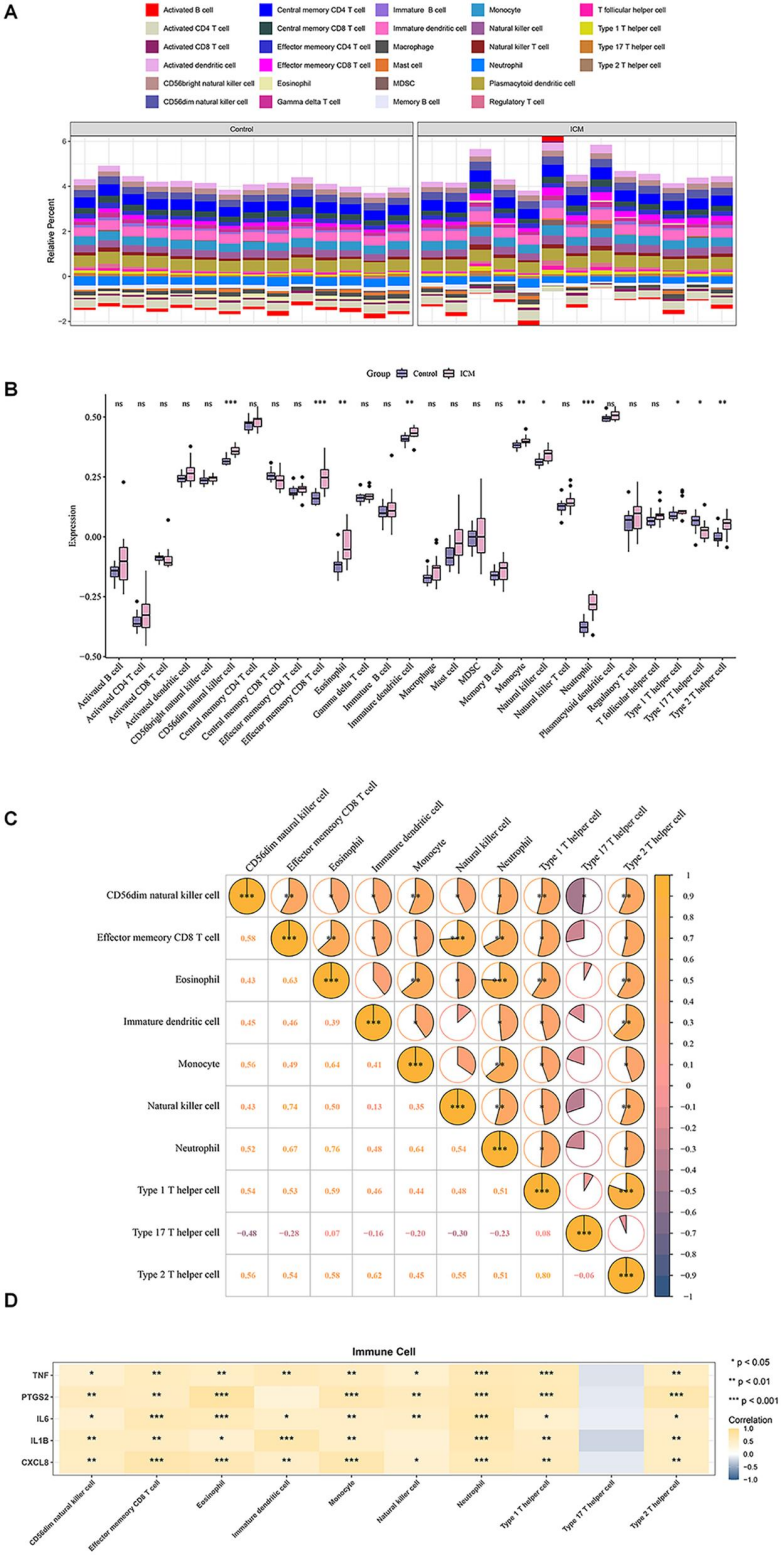
Immune cell infiltration analysis. **(A)** Distribution of 28 immune cell types in each individual sample. **(B)** Box plots showing differential infiltration of immune cells. **(C)** Correlation analysis of 10 significantly differentially infiltrated immune cell types. **(D)** Correlation analysis between hub genes and differentially infiltrated immune cells. *P < 0.05, **P < 0.01, ***P < 0.001.

By combining ssGSEA with the expression matrix from GSE116250,we assessed differences in immune cell infiltration between the control and ICM groups. The heatmap showed the distribution proportions of 28 immune cell types in each individual sample (Figure 4A). Among these 28 immune cell types,10 exhibited significant differences in ICM heart tissue,including CD56dim natural killer (NK) cells, effector memory CD8+ T cells, immature dendritic cells, neutrophils, type 1/type 2/type 17 helper T cells, etc. (Figure 4B). Meanwhile, except for Th17 cells, almost all significantly altered immune cell subtypes showed increased infiltration in the ICM groups compared with control group.

Furthermore, correlation analysis among significantly altered immune cell types between groups revealed CD56dim NK cell presents an extensive and significant correlations with other cells. In contrast, Th17 cell not significantly correlated with any other cell types, except for negative correlation with NK cells (Figure 4C). Correlations between the top 5 hub genes and 10 significantly different immune cell types exhibited that all the hub genes exhibited significant correlations with multiple immune cell infiltrates (Figure 4D).

### 3.5 Result of GSEA and GeneMANIA analyses

**Figure 5:**
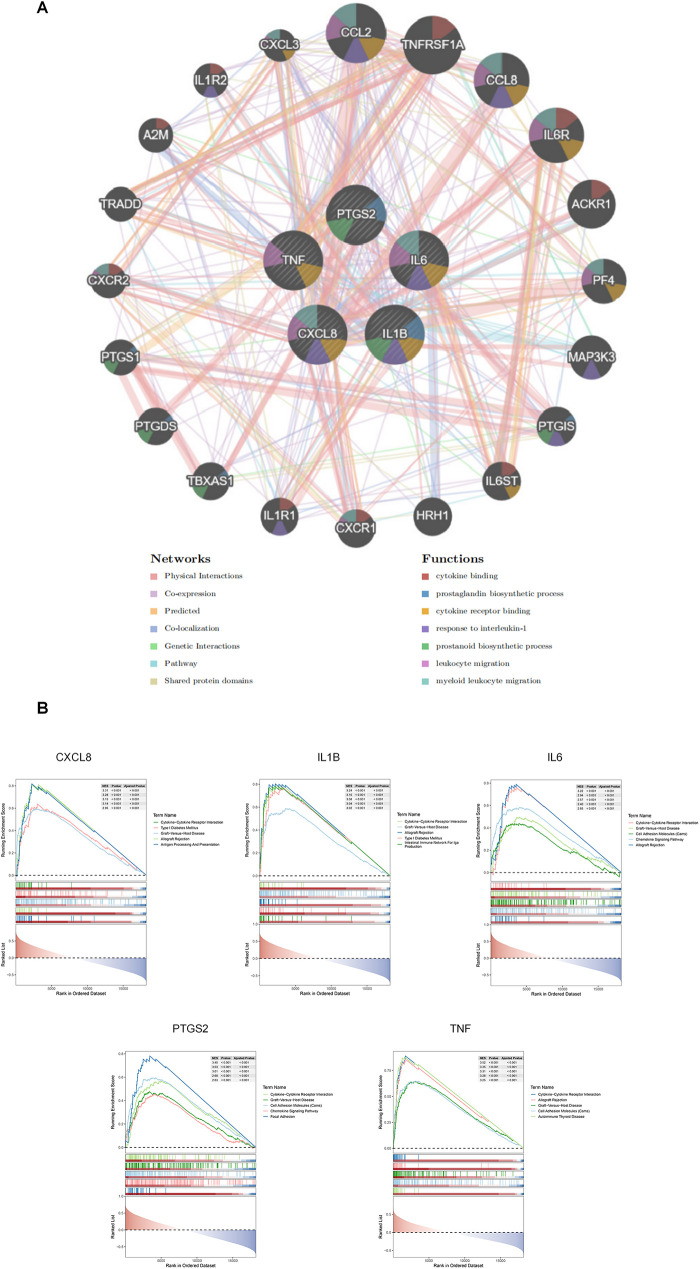
Results of GeneMANIA and GSEA analyses. **(A)** Results of GeneMANIA analysis: The color of nodes represents different protein functions, and the color of lines represents the type of interaction. **(B)** GSEA analysis results for the 5 hub ARDEGs.

GeneMANIA was used to predict the interacting genes of the hub genes, and a total of 20 genes predicted to interact with the hub genes were obtained. The hub genes were located in the inner circle, while the predicted genes were in the outer circle (Figure 5A). The functions of these predicted genes focused on cytokine binding, and correspondingly,the functions of the hub genes were concentrated on cytokine receptor binding.

This result was highly consistent with that of the GSEA analysis (Figure 5B), which indicated that all these hub genes were enriched in the cytokine-cytokine receptor interaction pathway. In fact, since the hub genes were well-known inflammatory cytokine genes from the same MCODE module,the functional enrichment results were predictable. However, these results clearly provide multiple potential receptor-blocking targets for alleviating the effect of aging-related hub genes on ICM.

### 3.6 Determination of regulatory signatures for 5 hub ARDEGs

**Figure 6:**
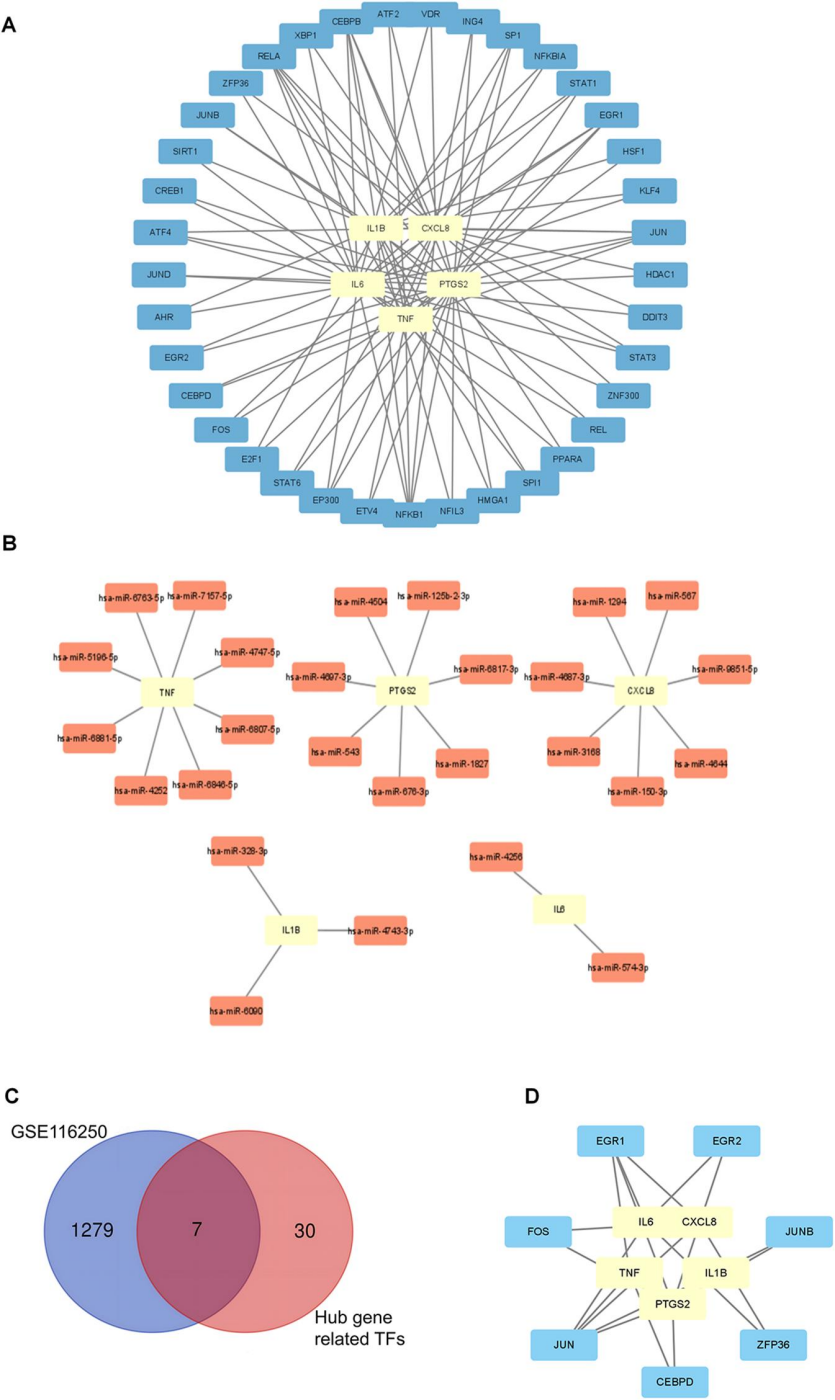
Transcription factors (TFs) -mRNA-miRNAs regulatory network analysis of ARDEGs. **(A)** TF regulatory network analysis of the top 5 hub ARDEGs. **(B)** miRNA regulatory network analysis of the top 5 hub ARDEGs. **(C)** Venn diagram of the differentially expressed genes analyzed based on GSE116250 and the predicted TFs. **(D)** The significantly expressed TFs in GSE116250 and their regulatory network prediction to the top 5 hub ARDEGs.

To gain insights into the regulatory molecules of the 5 hub ARDEGs and identify substantial changes at the transcriptional and post-transcriptional levels, we used publicly available bioinformatic databases to reveal potential TFs and miRNAs. A total of 37 related TFs were identified from the TRRUST database. Among these TFs, NF-kB1, CEPBP, RELA, and JUN had regulatory effects on all 5 hub ARDEGs (Figure 6A). We used the miRwalk and miRDB databases to predict the miRNA-mRNA regulatory network. The overlapping miRNAs from these two databases were regarded as target miRNAs and are depicted in Figure 6B. To further explore the potential TFs of ARDEGs in the ICM setting, we overlapped the 37 TFs with the GSE116250 dataset. As a result, 7 TFs were significantly elevated in the ICM group at the transcriptional level, as shown in Figure 6C. EGR1 was the most significant TF, with a 3.4-fold change in log2FC. JUN had the potential to regulate all 5 hub ARDEGs in GSE116250. Detailed information on these 7 TFs and their regulatory network with ARDEGs is presented in Table 3 and Figure 6D.

**Table 3:** A list of the 7 significantly expressed TFs in GSE116250.

Key TF	Description	Number of overlapped genes	List of overlapped genes	Expression in GSE116250 (Log2FC)
JUN	jun proto-oncogene	5	IL1B, CXCL8, PTGS2, TNF, IL6	Upregulated (1.27)
EGR1	early growth response 1	4	TNF, CXCL8, IL6, PTGS2	Upregulated (3.40)
EGR2	early growth response 2	2	PTGS2, IL6	Upregulated (3.36)
JUNB	jun B proto-oncogene	2	PTGS2, IL1B	Upregulated (2.02)
CEBPD	CCAAT/enhancer binding protein (C/EBP), delta	2	TNF, PTGS2	Upregulated (1.25)
FOS	FBJ murine osteosarcoma viral oncogene homolog	2	PTGS2, CXCL8	Upregulated (2.34)
ZFP36	ZFP36 ring finger protein	2	IL6, CXCL8	Upregulated (2.05)

### 3.7 Validation of hub ARDEGs based on human datasets

**Figure 7:**
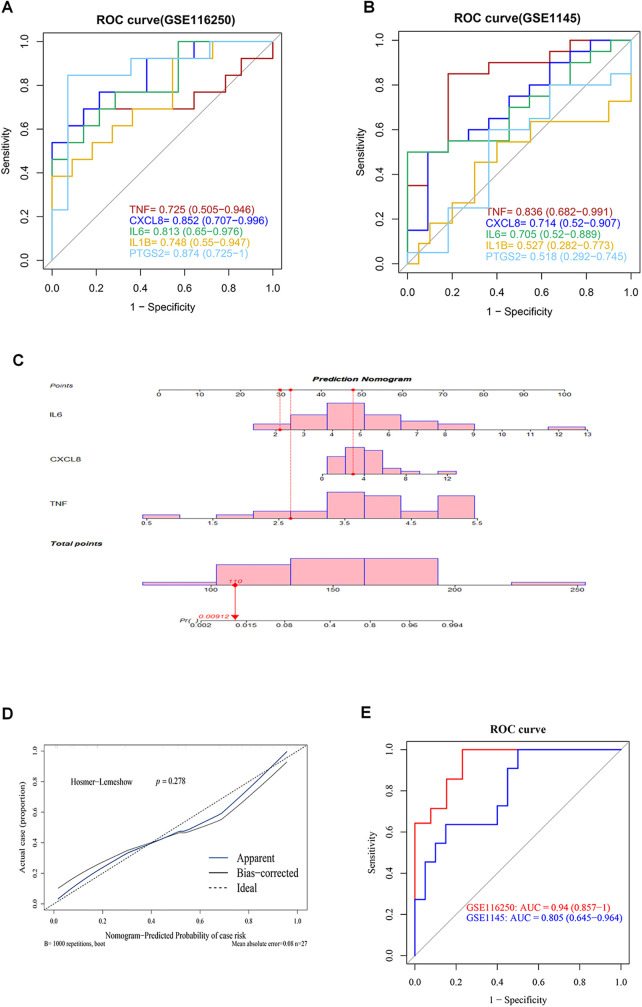
Validation of hub ARDEGs based on human datasets. **(A,B)** ROC analysis of the five hub ARDEGs in GSE116250 and the validation dataset (GSE1145) The *X*-axis represents the (1-Specificity), and the *Y*-axis represents the sensitivity. **(C)** Nomogram for ICM prediction. The frequency distribution is shown above the axis. **(D)** Calibration curve of the ICM prediction model. **(E)** ROC analysis of the three-gene combination in GSE116250 and validation dataset (GSE1145).

To evaluate the predictive value of these hub ARDEGs, we performed ROC analysis on both GSE116250 and the validation dataset GSE1145. The ROC curve revealed that the AUC values of all 5 hub genes in GSE116250 and 3 genes in GSE1145 (including TNF, CXCL8, and IL6) were ≥0.7 (Figures 7A,B). Subsequently, a nomogram was constructed using the expression levels of these three genes to predict ICM morbidity (Figure 7C), indicating that the combination of the three genes was effective in identifying ICM risk. The nomogram's predictive performance was then assessed: the apparent and ideal curves closely aligned in the calibration plot, with a *p*-value >0.05 from the HL test (Figure 7D). ROC analysis based on the three-gene combination demonstrated satisfactory predictive capability, with AUC values of 0.94 for GSE116250 and 0.805 for GSE1145, respectively (Figure 7E). These results suggested that the combination of these three hub ARDEGs has a potential predictive value for ICM.

### 3.8 Expression validation of hub ARDEGs in mouse MI model

**Figure 8:**
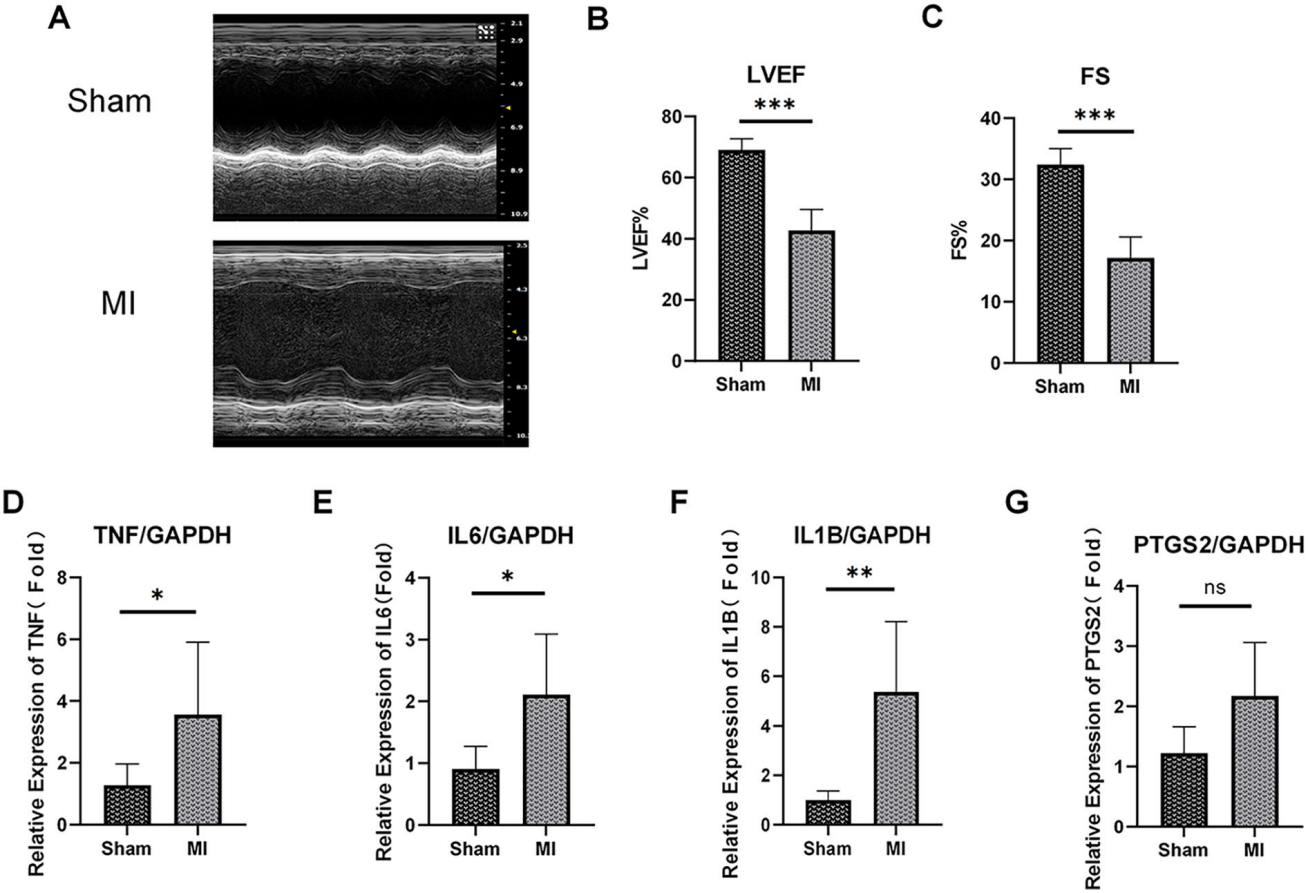
Expression validation of the top 5 hub ARDEGs on mouse MI model. **(A)** Representative M-mode echocardiography of ICM and control group at 1 week post MI. **(B,C)** Quantitative analysis of ejection fraction (EF) **(B)**, fraction shortening (FS) **(C)**. **(D–G)** The mRNA expression levels of TNF **(D)**, IL6 **(E)**, IL-1B **(F)** and PTGS2 **(G)** in the mouse heart (left ventricle) at 1 week post MI were determined by RT-qPCR assay. ns, not significant. ∗*p* < 0.05, ∗∗*p* < 0.01, ∗∗∗*p* < 0.001, *n* = 5-7.

To further validate the expression of these hub ARDEGs in ICM, we established an ICM animal model by ligating the coronary artery of aged mice. Echocardiograms were performed before ligation and 7 days after ligation to verify heart function changes. As shown in Figures 8A–C, mice in the MI group had significantly poorer cardiac function reflected as decreased FS and LVEF, indicating successful animal model construction. Then, RT-qPCR analysis was used to measure hub ARDEGs. Due to the absence of the human CXCL8 gene in rodents (1), we mainly measured the expression of the other four genes after MI. As expected, compared to the control group, the expression levels of *Il6*, *Tnf*, and *IL1b* were significantly upregulated in the MI group (*P* < 0.05, Figures 8D–F). *Ptgs2* also showed a tendency of significant expression, but there was no significant difference due to the degree of dispersion and the limitation of sample size (*P* = 0.07, Figure 8G).

## 4 Discussion

The incidence of ICM is increasing globally. Because of obstructive coronary artery diseases, it remains one of the leading causes of heart failure and heart transplantation (2). Importantly, most patients suffering from ICM show age-related changes of the heart and vessels accompanied by other chronic diseases such as hypertension, diabetes, metabolic disorder (3). All these co-morbidities are linked to aging-associated pathological mechanisms (3–6). Although the underlying mechanisms of ICM have been widely researched worldwide, effective therapies, especially for older patients, are still lacking. It is therefore crucial to identify novel targets specifically for ICM prevention and therapy.

The impact of aging on ICM is multifaceted. Firstly, aging is highly significant in the formation and progression of coronary atherosclerotic plaques, which form the basis for the development of obstructive coronary artery diseases. Multiple cell types (such as VSMCs, ECs, and inflammatory cells) exhibit marked changes during the aging process. Both prematurely and naturally aged cells show several characteristics, including changes in cell proliferative potential, cellular senescence markers, increased reactive oxygen species (ROS), a pro-inflammatory state, an increased propensity for cell death, elevated DNA damage, and extensive telomere shortening and dysfunction. All these features can be identified in cells from atherosclerotic plaques, which also display other features related to cell senescence (7). The underlying mechanisms of aging in AS are multiple and cumulative in any one cell or between cell types. Both aged ECs and aged VSMCs show a pro-inflammatory state, which enhances monocyte migration into the vessel wall (8). This is consistent with the significant increase of monocytes in the ICM tissue observed in the current study. Notably, aged VSMCs have been shown to secrete higher levels of IL6, which seems related to enhanced senescence-associated calcification in VSMCs (9). As an inflammation executor, aged monocytes exhibit an increased burst of free radicals upon activation and enhanced secretion of several cytokines, including IL6, TNF-a, IL1B, and monocyte chemotactic protein (MCP)-1, which also appear in AS (10). Thus, there are reasons to believe that aging accelerates pro- inflammatory changes in monocytes, leading to atherosclerosis. Under the combined effects of changes in adhesion molecules on aged ECs, as well as changes in VSMCs and monocytes, aging promotes the migration and activation of macrophages in plaques, thereby promoting the occurrence and development of atherosclerosis and ultimately leading to obstructive coronary artery diseases or even ICM.

In addition to its effect on AS, aging is also associated with the fate of cardiomyocytes during ischemia or ischemia-reperfusion (IR) injury. On one side, reactive oxygen species (ROS) generation following ischemia/IR injury is a factor in inducing pathological cardiomyocyte aging. Cardiomyocytes in an ischemic environment exhibit many aged-cell properties (11, 12). MI induces changes in mitochondrial dynamics, ultimately leading to elevated ROS production and enhanced oxidative stress, especially after the clinical treatment of reperfusion (13). Telomeres as the protective caps at the ends of chromosomes, are highly vulnerable to damage caused by ROS. This sensitivity is attributed to their guanine-rich regions, which are more prone to oxidation. The resulting oxidative stress can accelerate telomere attrition, leading to telomere dysfunction, premature senescence, and accelerated aging (14, 15). On the other hand, aged cardiomyocytes show more vulnerability during ischemia. Aged cardiomyocytes exhibit greater accumulation of Ca2+ during ischemia (16). Overloaded Ca2+ further impairs mitochondrial function and ultimately causes apoptosis (17). Apoptosis, the main cell death mode after MI, is closely associated with myocardial remodeling (18). Mounting evidence has shown that apoptosis occurs more often and seriously in aged cardiomyocytes during IR injury through various mechanism (19–21), and aged cardiomyocyte function is harder to recover from IR as well (16, 22). In addition to cardiomyocyte death, aging is also proved to impairs neovascularization, elevates inflammation, and facilitates the occurrence of serious arrhythmia under ischemia (23–25). Due to the combined effect of the above factors, aged hearts comparatively result in larger infarct size and worse cardiac function after ischemia (26), finally resulting in ICM.

In the current study, we conducted functional enrichment analysis to investigate the enriched GO items and significant pathways based on the ARDEGs. These ARDEGs are mainly related to response to inflammation and regulation of cell adhesion. These biological processes are consistent with the atherosclerotic plaque formation process and pathophysiological changes after myocardial infarction as above described. Among the significant findings from GO analysis, IL17A and NF-κB signaling pathways are potentially crucial in ICM development. IL17A, the best-studied member of the IL17 family which includes IL17A, IL17B, IL17C, IL17D, IL17E and IL17F, is secreted by various cells such as Th17 cells, *γδ* T cells, NKT cells, CD8T cells, macrophages, and dendritic cells. Thus, although Th17 cells were decreased in ICM according to the immune infiltration results, the significant elevation of other IL17-secreting cell types also effectively contributed to the activation of the IL17 pathway. In AS, increased total IL17A levels and expression in CD4T helper and *γδ* T cells have been demonstrated (27). IL17A mainly contributes to leukocyte accumulation, and it also affects factors like endothelial function and fibrous cap formation in plaque formation and is involved in damage or fibrosis process in myocardial tissue under MI (28). Meanwhile, the NF-κB family is activated in response to environmental and cellular stress, hypoxia, and ischemia, which initiates various pathological events like innate and adaptive immunity, cell survival, differentiation, and proliferation. It is a potential therapeutic target in AS and related CVDs. Multiple signaling molecules, including IL17, can activate NF-κB pathways in AS and MI (29). The NF-κB pathway is involved in different stages from plaque formation to its destabilization and rupture in AS and also in angiogenic and apoptotic processes under MI (30). Experimental studies show that inhibition of these pathways may reduce inflammatory burden, relieve AS development, and reduce cardiac damage after MI (31, 32).

IL6, TNF, IL1B, CXCL8, PTGS2 are the most important ARDEGs we conducted from this study. All of these genes are pro-aging genes which are elevated in ICM group, confirming that aging is an adverse factor in the occurrence and development of ICM. IL6, TNF -a and IL1B are well-studied genes in CVDs, which contribute not only to AS but also to cardiomyocyte death, cardiac function loss, malignant arrhythmia, neoangiogenesis, and myocardial remodeling after coronary occlusion. TNF-a, and IL1B are strong inducers of IL6 production. Many studies have demonstrated that the inhibition of TNF-a and IL1B is helpful in decreasing IL6 production during MI, and reduces infarct size, relieves cardiac remodeling, as well as improves cardiac function after MI (33–35). Meanwhile, clinical trials have shown that using tocilizumab to directly inhibit the activation of the IL-6 receptor reduces microvascular obstruction and improves prognosis in patients with MI (36). Therefore, the benefits of inhibiting TNF-a, IL1B and IL6 in MI and coronary AS may help limit the progression of MI to ICM.

PTGS2, better known as cyclooxygenase (COX) 2, is a mature target for the treatment of inflammatory pain in the clinical setting. Cox1 and Cox2 are COX-isozymes that vary in regulatory mechanisms of expression, tissue distribution, substrate specificity, preferential coupling to upstream and downstream enzymes, and susceptibility to inhibition by the highly heterogeneous class of COX-inhibitors. CoX-1 is constitutively expressed in most tissues such as blood vessels, stomach, kidney and platelets, and is involved in the regulation of platelet aggregation, vasomotor, gastric mucosal blood flow and renal blood flow to maintain the stability of physiological functions of cells, tissues and organs. The activity of COX-2 in normal tissue cells is extremely low. When stimulated by inflammatory factors such as IL1B, TNF-a, CD40l, the expression level of COX-2 in inflammatory cells can be sharply raised, resulting in an increase in the level of PGE2, PGI2 and PGE1 at the inflammatory site, leading to inflammatory response and tissue damage (37). In AS, COX-2 is expressed by macrophages, VSMCs and endothelial cells. It is crucial in the process of atherosclerotic plaque formation and plaque instability, thus further leading to the occurrence of atherothrombotic syndromes such as acute myocardial infarction (38). Compared to the clarity in AS, the effect of COX-2 in MI/I-R injury is controversial. Some studies support that COX-2 is a protective protein in ischemic injury (39–41), whereas others conducts an exact opposite conclusion (42–44). In fact, the apparent paradox of COX-2 inhibition can be reconciled through context-dependency. Specifically, the protective role of COX2 is primarily observed during the ischemic preconditioning phase. In this process, upregulation of COX-2 may alter the balance between thromboxane A2 (TXA2) and prostacyclin (PGI2), shifting it toward a protective phenotype. This improved balance helps mitigate the risks of vasospasm and thrombosis, thereby protecting myocardial tissue. Conversely, the benefit of COX-2 inhibition lies in its attenuation of the inflammatory response during ischemia. And the cardiovascular risks associated with COX-2 inhibitors are largely attributable to a TXA2/PGI2 imbalance, which promotes thrombotic events. ICM is characterized by persistent inflammation, progressive fibrosis, and adverse ventricular remodeling processes that develop over years. In the such chronic pathological milieu, our study confirms that COX-2 is highly expressed in ICM, which may accelerate AS, promote the occurrence of myocardial infarction, and exacerbate cardiac injury and fibrosis. It is worthy to believe that, appropriately inhibiting COX2 will yield benefits in ICM by reducing the inflammatory response. Therefore, targeting COX-2 in ICM should not be deny based on historical controversies but rather reevaluated with a refined strategy. Future efforts may focus on identifying the high inflammatory patient subpopulation to determin who would most benefit from COX-2 inhibition,while also optimizing the timing and dosing to mitigate chronic remodeling without impairing acute protective mechanism.

In comparison with others, CXCL8 seems to be less studied in both AS and MI.CXC chemokine ligand 8 (CXCL8), also recognized as neutrophil-activating peptide 1 (NAP1) and Interleukin-8 (IL-8) (45), is a proinflammatory CXC chemokine that plays a prominent role in inducing neutrophil chemotaxis, the release of intracellular granule contents, and the upregulation of cell surface adhesion molecules (46). According to reports, CXCL8 can participate in the occurrence and development of AS through multiple pathways such as promoting neutrophil extracellular traps (NETs) formation (47), promoting the proliferation and migration of VSMCs (48), inhibiting cholesterol efflux in foam cells (49), and regulating endothelial cell functions (50). Although CXCL8 has been widely demonstrated to elevated during MI (51–54), the role of CXCL8 in MI is still extremely limited. Perhaps due to the absent of CXCL8 expression in rodents that have been brought barriers to the research on the role and mechanism of CXCL8 in ICM (1). However, many bioinformatics analyses based on clinical data (53–55), including this study, identified CXCL8 as an important hub-gene in the occurrence and development of MI and AS. Therefore, more attention should be paid to CXCL8, and it should be regarded as a potentially important target for the diagnosis and treatment of ICM for further research. Lastly, we conducted some miRNA and transcription factors which are potential upstream regulated factors of these five hub-ARDEGs by using bioinformatic method. We then verified the expression of these transcription factors in GSE116250. It is worth noting that JUN was identified as a key TF that was elevated in ICM and showed the ability to regulate all the five hub-ARDEGs expressions in this study. Hence, it deserves more study to investigate its effect on aging related pathway in ICM. Gain/loss-of-function studies may be required to further confirm its regulatory effect on these hub genes.

Despite the fact that IL6, TNF, IL1B, CXCL8 and PTGS2 expression enhanced have been confirmed in AS, MI and ICM based on many studies (33–35, 39–44, 52–55), the predicted value of PTGS2 and IL1B in the GSE1145 dataset was depressing in this study. It is probable that because ICM samples were mainly obtained from the heart transplantation recipients, limited sample size contributed to the results' bias. Additionally, the different locations of samples used for the experiment (e.g., ischemic zone or ischemic marginal area) and the different disease stages of ICM may also cause gene expression heterogeneity. In contrast, IL6, TNF, and CXCL8 exhibited promising predictive value for ICM. The combination of these three genes showed satisfactory predictive performance in both GSE116250 and GSE1145. However, although IL6, TNF, and CXCL8 encode secretory cytokines that may be detectable in the blood, their non-invasive predictive value for ICM still requires further investigation.

Lastly, the complex mechanism of ICM also brought some inevitable flaws to this study. ICM involves multiple pathological processes including AS, MI, and cardiac remodeling. The simple mouse MI model with ligation of the LAD is incapable of fully mimicking the entire process from coronary atherosclerosis formation to myocardial infarction and ultimately to cardiomyopathy during ICM. This limitation may have led to the non-significant expression of *Ptgs2*. However, since *Ptgs2* already showed a trend toward differential expression, increasing the sample number, performing analyses at the protein level, or adding more time points may allow the observation of significant differences in PTGS2 expression in subsequent validation experiments. Furthermore, due to the CXCL8 gene absence in the mouse genome, its expression was not verified in our animal experiment which caused an insufficient of this study. The limited availability of normal human hearts as a control group restricted our ability to perform experimental verification in humans, but it is certain that testing human samples would provide a more robust validation to address these limitations. Overall, to further explore the in-depth mechanism of ARDEGs in ICM, more specific and rigorous experiments should be designed and executed in future research.

## 5 Conclusion

In summary, this study revealed that the immune infiltration profile and expression pattern of aging-related genes in ischemic cardiomyopathy (ICM) differ significantly from those in normal tissues. A total of 50 dysregulated aging-related differentially expressed genes (ARDEGs) and their associated pathways were identified in ICM. Protein-protein interaction (PPI) analysis highlighted the top five most critical ARDEGs, including IL6, TNF, CXCL8, PTGS2, and IL1B, which were further validated. These hub ARDEGs were significantly upregulated in the myocardial infarction (MI) context and may appear to play a pivotal role in the initiation and progression of ICM. Among them, IL6, CXCL8, and TNF exhibited a predictive value for ICM. Consequently, these hub ARDEGs may serve as potential signature genes for ICM.

This study underscores the critical role of aging in ICM and suggests that targeting key ARDEGs, such as TNF, PTGS2, IL6, IL1B, and CXCL8, may provide promising diagnostic and (or) therapeutic avenues for ICM management.

## Data Availability

Publicly available datasets were analyzed in this study. This data can be found here: Gene Expression Omnibus (GEO) repository (https://www.ncbi.nlm.nih.gov/geo/), accession numbers: GSE116250 and GSE1145.
