# Supplementary material for: Comprehensive analysis of aging-related genes and immune infiltration landscape in ischemic cardiomyopathy
Source: Front Cardiovasc Med. 2025 Sep 11;12:1653314. doi: 10.3389/fcvm.2025.1653314 (PMC12461871; doi:10.3389/fcvm.2025.1653314)
Supplement: Supplementary file 1 [file Table1.docx]

#### Supplementary Table 1. Primer Sequences for Validating Gene Candidates.

| Gene Name | Primer Sequence (5’-3’) | |
| --- | --- | --- |
|  | Forward Primer | Reverse Primer |
| *Il6* | TAGTCCTTCCTACCCCAATTTCC | TTGGTCCTTAGCCACTCCTTC |
| *Tnf* | CCCTCACACTCAGATCATCTTCT | GCTACGACGTGGGCTACAG |
| *Il1b* | CCTTCCAGGATGAGGACATGA | TGAGTCACAGAGGATGGGCTC |
| *Ptgs2* | TGAGCAACTATTCCAAACCAGC | GCACGTAGTCTTCGATCACTATC |
| *Gapdh* | TGCACCACCAACTGCTTAG | GGATGCAGGGATGATGTTC |
